# Efficacy of a Mobile Health App in Promoting Breastfeeding and Young Child Feeding Practices in Pakistan: Protocol for a Randomized Controlled Trial

**DOI:** 10.2196/93181

**Published:** 2026-07-20

**Authors:** Rozina Nuruddin, Rehama Iqbal, Nuruddin Mohammed, Khadija Barkat Ali Vadsaria, Sidrah Nausheen, Saleem Sayani, Iqbal Azam

**Affiliations:** 1 Department of Community Health Sciences, Medical College, Aga Khan University, Stadium Road, Karachi, Sindh, 74800, Pakistan, 92 21 3486 483, 92 21 3493 4294; 2 Medical College, Aga Khan University, Karachi, Sindh, Pakistan; 3 Department of Obstetrics and Gynecology, Medical College, Aga Khan University, Karachi, Sindh, Pakistan; 4 Digital Health Resource Center, Aga Khan University, Karachi, Sindh, Pakistan; 5 Department of Community Health Sciences, Medical College, Aga Khan University, Karachi, Sindh, Pakistan

**Keywords:** Infant and young child feeding practices, exclusive breastfeeding, complementary feeding, mobile health app, malnutrition

## Abstract

**Background:**

In Pakistan, one-third of under-5 deaths (58/1000 live births) are attributable to the high prevalence of stunting (40%), underweight (23%), and wasting (17.7%). Given the suboptimal prevalence of exclusive breastfeeding (EBF; 48%) and the alarmingly low consumption of a minimum acceptable diet (3.6%), mitigation of early-life nutritional risk provides a critical window of opportunity for intervention. Mobile health (mHealth) provides an innovative and low-cost option to improve infant and young child feeding practices (IYCFPs) among mothers.

**Objective:**

This study aims to assess the efficacy of a context-specific mHealth coaching app in promoting EBF and IYCFPs compared to face-to-face (F2F) counseling.

**Methods:**

This is a prospective, parallel-arm randomized controlled trial planned at a secondary care hospital in Karachi, Pakistan. The study will enroll 300 booked singleton pregnant women in the third trimester, who plan to stay in their respective areas for at least 1 year postdelivery, are registered for child immunization at the associated Family Health Center, own smartphones with internet access, are able to read Urdu, and provide consent. Participants in the intervention arm will receive the *Pehli Ghiza* mHealth app along with routine standard-of-care F2F counseling. The app will deliver context-specific educational content on breastfeeding and IYCFPs through short videos and messages sent 3 times per week, tailored to the stage of pregnancy and infant age. Participants in the control arm will receive only routine F2F counseling. The standard of care will be delivered at each follow-up visit, which is scheduled at birth, at 6, 10, and 14 weeks, and at 6, 9, and 12 months of the infant’s age. Data will be collected at enrollment and at each follow-up visit using structured questionnaires. Primary outcomes are EBF and the introduction of age-appropriate complementary feeding. Secondary outcomes include early initiation of breastfeeding, continued breastfeeding at 1 year, minimum dietary diversity, meal frequency, minimum acceptable diet, and child health outcomes. Analysis will follow the intention-to-treat principle to detect a 20% absolute improvement in primary outcomes between the intervention and control groups over time and to evaluate between-group differences in feeding practices. Compliance will be determined by the proportion of participants who complete the 6-month coaching program. Usability will be assessed based on features related to design, interface, content, coaching, perception, and personal benefit.

**Results:**

The study was approved by the Ethics Review Committee of the Aga Khan University and the National Ethics Committee. Participant recruitment started on February 2, 2026. As of May 18, 2026, 241 participants have been recruited. Follow-ups and outcome assessments are expected to be completed by June 2027.

**Conclusions:**

If effective, the *Pehli Ghiza* intervention could support the integration of digital health tools into standard of care in Pakistan and similar low- and middle-income country settings.

## Introduction

Undernutrition remains a critical global health issue, contributing to approximately 35% of the global disease burden [1] and accounting for 11% of all disability-adjusted life years [1]. It is associated with nearly half of all deaths among children younger than 5 years in low- and middle-income countries (LMICs) [2]. In Pakistan, the prevalence of stunting among children younger than 5 years remains alarmingly high at 40.2% [3], nearly double the regional average of 21.8% [4]. Wasting has increased from 8.6% (1997) [3] to a peak of 17.7% (2018) [3], significantly surpassing the World Health Organization (WHO) threshold of ≥5% for a public health emergency [5]. These deficits are reflected in the country’s high infant (50/1000 live births) [6] and under-5 (58/1000 live births) [6] mortality rates. Beyond health, the economic burden of undernutrition in Pakistan is substantial, costing an estimated 3% to 4% of the national gross domestic product due to lost productivity, increased health care expenditures, and diminished human capital [7].

Evidence shows that optimal infant and young child feeding practices (IYCFPs) are among the most effective interventions to prevent malnutrition and improve child survival [8]. These refer to early initiation of breastfeeding (EIBF) within 1 hour of birth, exclusive breastfeeding (EBF) for the first 6 months of life, and introduction of adequate and safe age-appropriate complementary foods (AACF) at 6 months, together with continued breastfeeding up to 2 years of age [9].

Despite modest progress, significant gaps persist in Pakistan’s infant and young child feeding (IYCF) indicators. The recent National Nutritional Survey reported improvements in EIBF from 40% (2011) [10] to 45% (2018) [3]; yet, nearly 40% of neonates continue to receive prelacteal feeds [3], undermining the benefits of colostrum feeding. Further, a nonlinear trend was found in EBF, decreasing from 50% (2001) [3] to 37.7% (2011) [10] before rising to 48% (2018) [3], reflecting inconsistent policies and nonsustainable interventions. The decline in EBF was particularly evident at 3 months of infant age, dropping from 84% to 70% [11], reportedly due to inadequate maternal knowledge about EBF and its associated benefits [12]. The continued breastfeeding practice at 1 year also declined from 77.3% (2011) [10] to 68.4% (2018) [3], with only 40.1% of children aged 0 to 23 months receiving age-appropriate breastfeeding [3]. This means that while initial breastfeeding practices may be improving, their maintenance and adherence remain inadequate and challenging. Further, the complementary feeding (CF) indicators were even more concerning. Only 36% of children aged 6‐8 months receive timely CF; minimum dietary diversity (MDD) is achieved by 14%, minimum meal frequency (MMF) by 18%, and a mere 3.6% of children meet the minimum acceptable diet (MAD) criteria [3], a composite indicator reflecting both meal frequency and diversity. These significant deficiencies impair infant physical growth, immunity, and cognitive development.

This alarming situation has not improved much, as a recent study conducted in rural Sindh Province reported that <10% of infants were EBF, with a significant proportion identified as wasted (14.7%), stunted (36.7%), and underweight (38.5%) at birth [13]. The study also found a significant association of early initiation of CF or breast milk alternatives before 4 months and increased odds of wasting by 6 months of age (adjusted odds ratio 4.14, 95% CI 1.95-8.77; *P*<.001) [13]. This further reflects persistent gaps in early child nutrition, which are the result of a complex interplay of inadequate maternal knowledge, sociocultural norms and myths, and insufficient nutritional counseling at health system touchpoints [14-16].

Addressing these gaps requires scalable, contextually appropriate, and sustainable strategies extending beyond established practices. The traditional approaches relying on face-to-face (F2F) nutritional counseling, whether facility-based or through community health workers, remain resource-intensive and face challenges of reach, quality, and sustainability. In this regard, mobile health (mHealth) interventions are promising tools, particularly for LMICs with limited health care resources [17]. Pakistan has experienced rapid digital expansion. Household internet access has increased from 34% (2018) to 70% (2025) [18], and individuals using the internet have increased from 17% (2018) to 57% (2025) [18]. Smartphone penetration has risen from 10% (2014) [19] to 64% (2024) [20], while 96% of households reported ownership of a mobile phone or smartphone [18], corresponding to 196 million cellular subscribers [21]. The majority of these users (77%) are young adults aged 21 to 30 years [22], representing a critical entry point for digital health interventions among this receptive population.

Studies have been conducted to evaluate mHealth in promoting optimal feeding practices. A mobile-based approach among teenage mothers in Nigeria reported an increase in knowledge scores about IYCFPs from 38.7% to 76.1% and in EBF practice from 49.3% to 63.5% at 6 weeks postintervention [23]. An mHealth intervention in Nepal improved maternal IYCF knowledge scores (β=.21, 95% CI 0.08‐0.35) and dietary diversity, primarily for egg consumption (odds ratio 1.36, 95% CI 1.07‐1.73) [24]. In Indonesia, an mHealth intervention resulted in a 1.45-fold increase in overall IYCF knowledge [25]. In rural Pakistan, a 14.8% increase in overall IYCF knowledge delivered via text messages was reported among mothers [26]. A prospective cross-sectional survey to evaluate the utility of *LactApp*, a mHealth app about breastfeeding, showed that users were satisfied (87.8%), felt assisted (75.3%), and supported (80.9%) in their experience [27].

However, these studies are limited by a predominant focus on knowledge enhancement rather than sustained behavioral change or long-term feeding practices. Evidence regarding the effectiveness of mHealth interventions on clinically meaningful IYCFPs remains inconsistent. For instance, a cluster randomized controlled trial (RCT) conducted in South Ethiopia demonstrated a significantly higher probability of maintaining EBF at 6 months in the intervention group compared to the control group (adjusted hazard ratio 0.40, 95% CI 0.26‐0.62; *P*<.001) [28]. Similarly, a quasi-experimental Australian study evaluating the Growing Healthy infant feeding app reported reduced health service utilization among app users, including lower odds of using one or more health services (odds ratio 0.38, 95% CI 0.25‐0.59), fewer visits to general practitioners (1.0 vs 1.5 visits; *P*=.003), and fewer pediatrician visits (0.3 vs 0.4 visits; *P*=.049), although no significant differences in out-of-pocket costs were observed [29]. Conversely, another RCT conducted in the United States evaluating the Breastfeeding at Augusta University smartphone app found no statistically significant differences in breastfeeding duration or breastfeeding self-efficacy between the intervention and control groups [30]. Mothers in the intervention arm breastfed for 10.1 (SD 3.5) months compared with 8.9 (SD 4.1) months in the control arm (*P*=.32), while antepartum and 1-year postpartum breastfeeding self-efficacy scores also did not differ significantly (*P*=.96) [30]. These contrasting findings suggest that the effectiveness of mHealth interventions may depend on specific intervention components, contextual adaptation, engagement strategies, and the intensity of participant interaction.

Systematic reviews further highlight persistent evidence gaps. Reviews of studies conducted in LMICs have reported inconclusive evidence regarding CF practices, dietary diversity, and sustained IYCF outcomes [31-33]. Another systematic review and meta-analysis evaluating digital health interventions versus standard care for improving EBF duration among postpartum mothers in LMICs identified substantial heterogeneity across studies (*I*²=77%) in intervention components, study designs, and outcome measures [34]. Collectively, these findings underscore the limited availability of rigorous, contextually tailored, and longitudinal evidence regarding the causal effectiveness of mHealth interventions in improving sustained breastfeeding and IYCFPs, particularly in low-resource settings.

In Pakistan, while cross-sectional surveys have examined the use of mobile technology in the broader maternal and child health domain [35-38], no RCT has rigorously evaluated the effectiveness of a culturally tailored mHealth intervention on sustained IYCFPs. Consequently, there is a lack of high-quality evidence to inform the integration of digital tools into national nutrition programs. Given Pakistan’s high burden of undernutrition, suboptimal feeding practices, and expanding digital infrastructure, this study aims to evaluate the efficacy of a culturally tailored mHealth coaching app, *Pehli Ghiza* (“first diet”), in promoting EBF and age-appropriate IYCFPs among mothers during the first year of infant life, generating actionable evidence for national nutrition strategies and policy in Pakistan and similar LMIC settings.

## Methods

### Study Design

**Figure 1.**
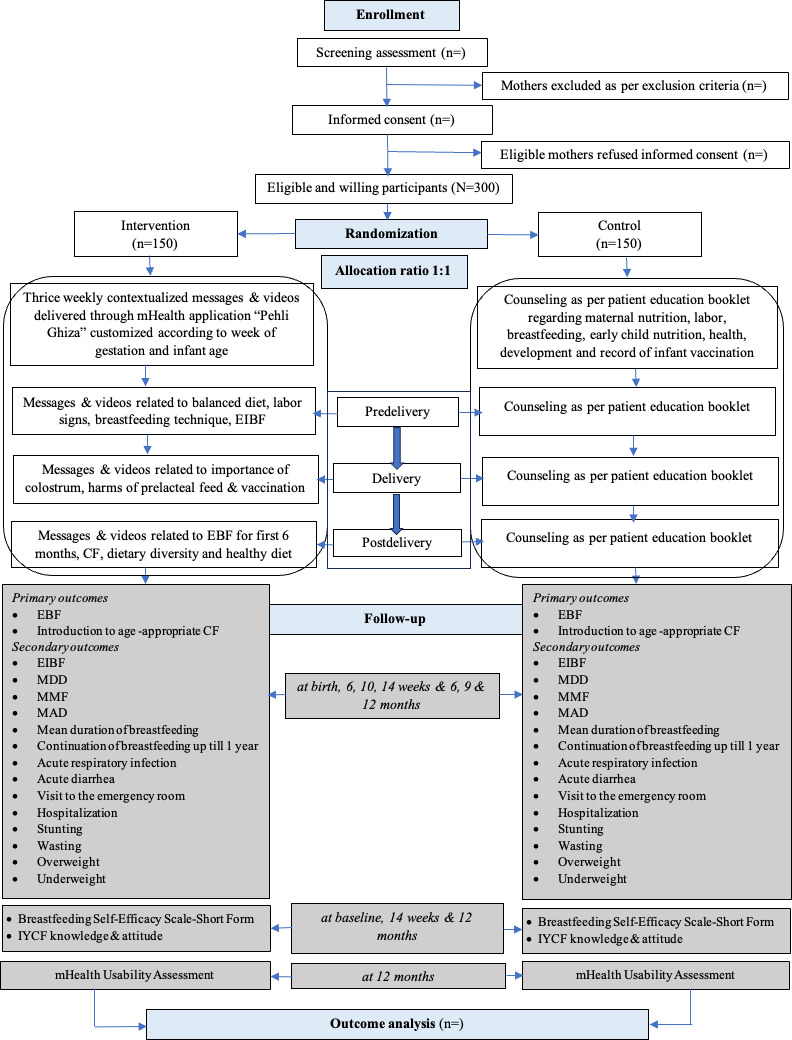
CONSORT (Consolidated Standards of Reporting Trials) flow diagram of the study. CF: complementary feeding; EBF: exclusive breastfeeding; EIBF: early initiation of breastfeeding; IYCF: infant and young child feeding; MAD: minimum acceptable diet; MDD: minimum dietary diversity; MFF: minimum meal frequency; mHealth: mobile health.

This is a 2-arm, parallel-group, superiority RCT with random allocation in a 1:1 ratio to the intervention and control groups (Figure 1).

### Study Setting and Duration

The trial will be conducted at the Women and Child Secondary Care Hospital, affiliated with the Aga Khan University Hospital (AKUH), located in the Kharadar area of Karachi, Sindh. The trial is planned to commence in February 2026 and will likely end in May 2027.

### Study Participants

#### Inclusion Criteria

Inclusion criteria were as follows: women aged 18 years or older, at 36±1 weeks of gestation, with a singleton pregnancy without risk factors, registered for delivery at the study sites, planning to stay in their respective areas for at least 1 year postdelivery and to register for child immunization at associated Family Health Centers, owning smartphones with internet access, able to read Urdu, and providing consent for participation.

#### Exclusion Criteria

Exclusion criteria were as follows: high-risk pregnancies, including maternal neurological, heart, autoimmune, or renal disease, preeclampsia, placenta previa, multiple gestations (twins/triplets), fetal structural or genetic anomalies, fetal growth restriction, and birth trauma requiring neonatal intensive care unit admission, women who require referral, and women who plan to move to a different residential area after delivery.

### Sample Size

The sample size was calculated to detect a 20% absolute difference in the primary outcomes between the intervention and control arms. The calculation assumed a baseline prevalence of EBF of 48% [3] and AACF of 50% [39] in the control group. Using a 2-sided significance level of 5%, statistical power of 80%, a 1:1 allocation ratio, and an anticipated 20% loss to follow-up in each study arm, a total sample of 300 pregnant women (150 per arm) was determined to be sufficient to detect the expected intervention effect. Loss to follow-up included participant withdrawal and/or discontinuation of app use in the intervention arm. Sample size estimation was performed using *OpenEpi* software (version 3.0).

### Recruitment

Participants who have given consent will be recruited through purposive sampling by the research assistant (RA). Informed consent will be taken in a private room at the obstetric clinics. Purposive sampling will ensure that participants meet the technology-specific eligibility criteria, which are essential for valid effect estimation [40]. Importantly, randomization will occur after purposive recruitment to preserve internal validity.

### Randomization and Allocation Concealment

Participants will be randomly assigned in a 1:1 ratio to the intervention or control arm using a computer-generated block randomization sequence prepared by the AKUH Clinical Trials Unit. Allocation concealment will be ensured using opaque, sealed, and sequentially numbered envelopes containing group assignments. The envelopes will be opened sequentially after participant enrollment to ensure concealed allocation and minimize selection bias.

### Blinding and Bias Minimization

Due to the nature of the intervention, blinding of participants and the RA is not feasible. To minimize observer and reporting bias, standardized outcome assessment procedures will be implemented across both study arms. The RA will receive training on neutral interviewing techniques and adherence to standardized counseling scripts to reduce differential probing or influencing participant responses. Structured questionnaires will be used to ensure consistency and reduce subjective interpretation during outcome assessment. In addition, data analysis will be conducted using coded group allocation to minimize analytical bias.

### Intervention

#### Pehli Ghiza (First Diet) mHealth App

Participants randomized to the intervention arm will receive educational awareness through the Pehli Ghiza mHealth app. This is in addition to routine standard-of-care F2F counseling delivered to all participants in both arms at each follow-up visit. The inclusion of standard of care will allow for the evaluation of the incremental effect of the intervention, such that any observed between-group differences can be attributed to the intervention rather than to the standard of care.

#### Theoretical Framework

**Figure 2.**
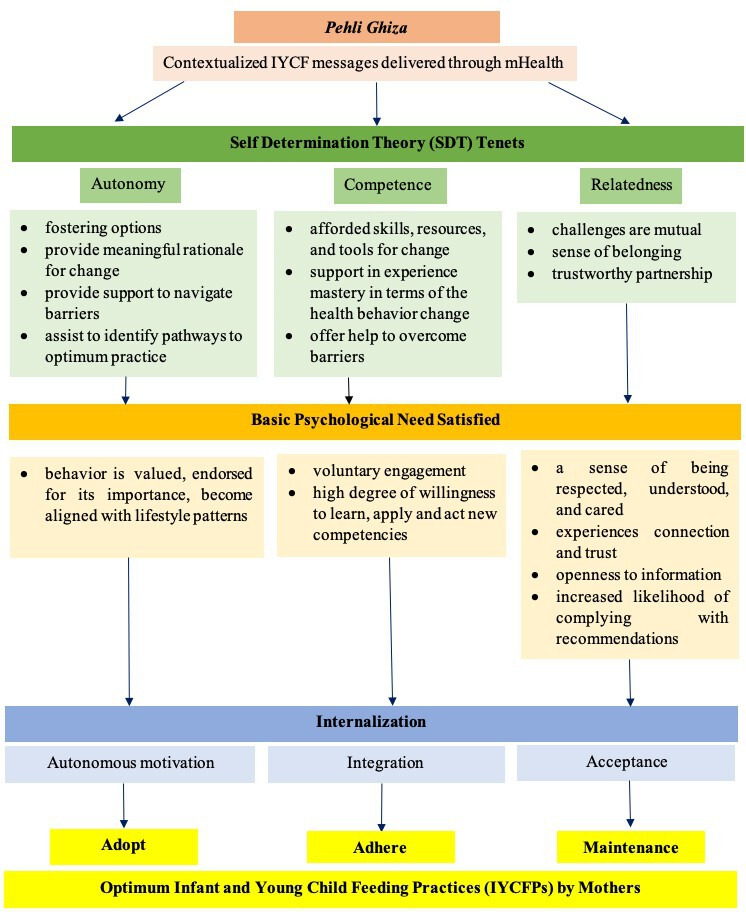
Theoretical framework summarizing the application of pillars of self-determination theory to the development of mobile health content. IYCF: infant and young child feeding; mHealth: mobile health.

The intervention design and messaging were informed by self-determination theory [41], which posits that sustained behavior change is facilitated when 3 basic psychological needs of autonomy, competence, and relatedness are supported. Fulfillment of these psychological needs promotes internalization and autonomous motivation, thereby increasing the likelihood of adopting, adhering to, and maintaining optimal IYCFPs over time (Figure 2). The messages were designed to align with these constructs (Table 1). *Autonomy* was addressed by providing mothers with a meaningful rationale for recommended IYCFPs, practical options, and support to overcome feeding barriers. *Competence* was supported through skill-based educational content, videos for practical guidance, and reinforcement reminders aimed at improving mothers’ confidence and ability to adopt optimal feeding practices. *Relatedness* was promoted by delivering supportive and culturally sensitive messages intended to foster trust, understanding, and a sense of connection with the intervention.

**Table 1.** Examples of infant and young child feeding practices messages aligned with self-determination theory constructs of autonomy, competence, and relatedness.

Feeding stage	mHealth^a^ messages^b^	Objectives	Autonomy	Competence	Relatedness
Pregnancy	What you eat lays the foundation of a healthy child. Eat fresh fruits and vegetables, drink plenty of water, and avoid excess consumption of tea, coffee, and processed food Do not forget, breastfeeding is the best gift a mother can give to her newborn Breastfeeding can lead to long-term health benefits for infants, such as a reduced risk of asthma, obesity, and type 1 diabetes	Generate willingness to improve and maintain healthy dietary habits and motivation for breastfeeding during pregnancy	Getting an insight into one’s own current personal dietary choices in an autonomous way, with little external control	Feeling confident to change diet patterns	Reflect upon dietary practices
EIBF^c^	Do not waste the yellow milk produced soon after delivery. Feed it to the baby, as it contains essential nutrients and protection against diseases.	Foster motivation to perform EIBF	Self-awareness without external control about the importance of EIBF	Feeling confident toward learning the skill of breastfeeding	Being at ease to learn the breastfeeding skill
EBF^d^	Your baby needs only breast milk for the initial 6 months, as it fulfills all food, water, and energy requirements It is important to drink water regularly and eat healthy food like eggs, meat, lentils, yogurt, milk, fresh fruits, and vegetables. It keeps you healthy, strong, and improves your milk flow	Develop commitment to maintain EBF for 6 months Create mindfulness regarding the importance of maternal diet while nursing	Provide self-confidence to perform and maintain EBF in an autonomous way, not imposed	Feel confident to maintain the learned behavior of EBF	Ready to improve breastfeeding skill
CF^e^ (6 to 12 mo)	Your baby, at 6 mo of age, requires healthy foods in addition to breast milk to grow and develop Continue breastfeeding on demand both day and night Begin with the staple foods like porridge (wheat, rice), mashed banana, or mashed potato Give 2 to 3 tablespoonfuls to taste at each feed Give food at least 2 times a day	Expand readiness to establish CF while maintaining an intention to continue breastfeeding till 2 years of age	Develop a plan to best maintain breastfeeding along with CF independently, without coercion	Feel confident to initiate a healthy complementary diet and maintain breastfeeding	Reflect to learn, improve, and maintain optimum CF practices

a mHealth: mobile health.

b Messages are adapted from [42].

c EIBF: early initiation of breastfeeding.

d EBF: exclusive breastfeeding.

e CF: complementary feeding.

#### Content Development

Initial formative research with the target population informed the design of the *Pehli Ghiza* mHealth app and guided the contextual adaptation and appropriateness of its content and delivery to locally identified barriers and facilitators related to IYCFPs. The educational content, based on WHO and UNICEF (the United Nations Children’s Fund) IYCF guidelines [43-45] and AKUH educational materials [46], focused on maternal nutrition, breastfeeding, CF, child immunization, and early growth and development. Specific and brief messages, tailored according to maternal gestational stage and infant age, were developed for dissemination through the mobile app. Messages were initially developed in English, translated into Urdu, and subsequently back-translated to ensure linguistic accuracy and consistency. The messages were reviewed and content validated by lactation and nutrition experts for scientific accuracy, clarity, and contextual appropriateness. The intervention messages and app interface were pilot tested among the target population prior to implementation, and participant feedback was incorporated to improve usability and acceptability.

#### Messages Delivery Format

**Figure 3.**
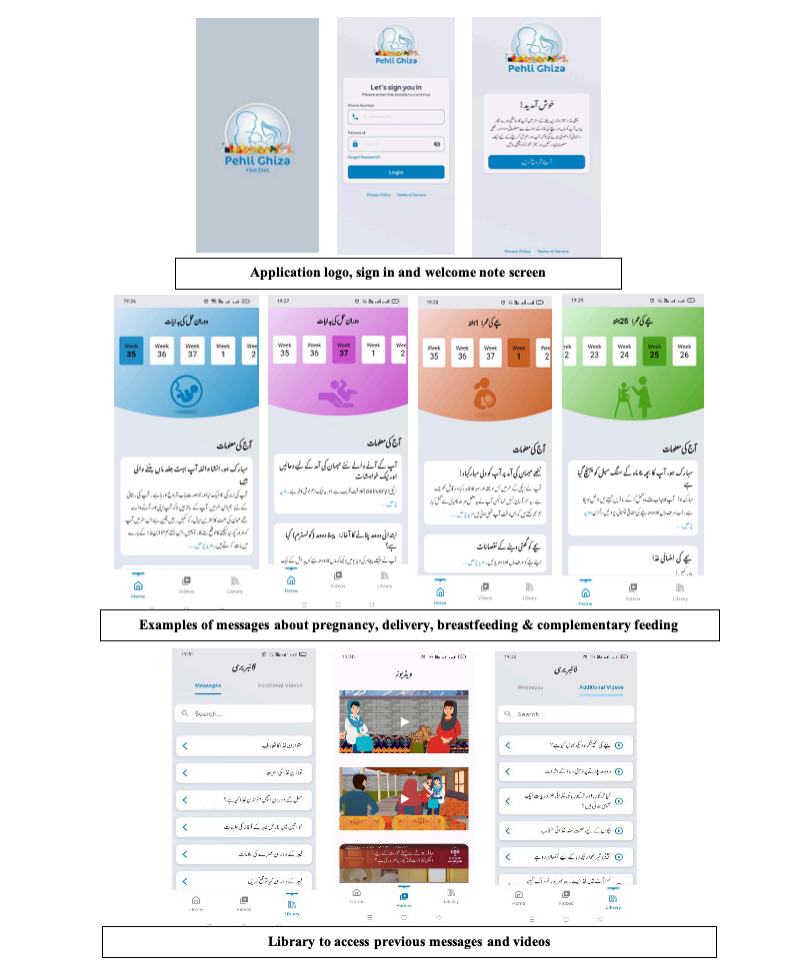
*Pehli Ghiza* (First Diet) mobile health app screenshots showing the logo, sign-in credentials, welcome note, messages contextualized according to the predelivery, delivery, and postdelivery stages, and library access to archived messages and videos.

Tailored educational messages, adapted to maternal gestational stage and infant age, will be delivered in the form of short text messages and brief videos of approximately 2 minutes in duration (Figure 3). This format is designed to maintain attention, enhance engagement, and reduce cognitive burden among participants.

#### Message Schedule

Messages will be delivered 3 times per week, beginning in the predelivery period and continuing until the child reaches 12 months of age. This schedule ensures continuous exposure to age-appropriate information during pregnancy and early infancy.

#### Application Onboarding

At enrollment, the RA will install the app on the smartphones of participants randomized to the intervention arm and provide a hands-on demonstration of its functionality to ensure correct initial use. To further optimize intervention adherence, participants will receive automated in-app notifications informing them of newly released content, along with reminder messages if content remains unaccessed for 48 hours. These prompts are intended to minimize missed exposure and sustain consistent engagement with the intervention over time.

#### Intervention Adherence Optimization

In addition, adherence optimization will be reinforced through active monitoring and follow-up. The RA will conduct fortnightly contacts to assess app performance, identify barriers to use, and provide tailored support to improve continued engagement, thereby strengthening intervention adherence throughout the study period. Technical troubleshooting will be supported by the Digital Health Resource Center team. At each follow-up visit, the RA will verify app functionality and reinstall the app if required.

#### User Engagement Metric

A set of engagement indicators will be derived from *MixPanel* mobile analytics [47], including video view counts, module completion status, time spent on each module, and frequency of app access. These metrics will provide an objective, time-stamped record of participant interaction with the intervention, enabling assessment of both the intensity and pattern of exposure over time.

#### Intervention Fidelity Assessment

The user engagement indicators will be used to assess intervention fidelity by evaluating the extent of participant exposure and adherence to the intervention as intended. Fidelity will be evaluated through cumulative exposure to scheduled content, the proportion of modules completed, and the average duration of interaction with intervention components. Based on predefined thresholds, participants will be categorized into fidelity groups as high (≥70% of scheduled content viewed and ≥70% of modules completed), moderate (40%‐69% of scheduled content viewed), and low (<40% of scheduled content viewed) fidelity [48]. These fidelity classifications will allow examination of dose-response relationships between intervention exposure and primary outcomes and will help interpret the effectiveness of the intervention in relation to varying levels of engagement.

#### Technical Architecture

*Pehli Ghiza* was developed with support from the Digital Health Resource Center of the Aga Khan Development Network. The app development stack includes the Flutter software development kit (SDK; version 3.38) for cross-platform, high-performance development; the Dart SDK bundled with Flutter 3.38 programming language; the Android SDK latest stable (API level 35) for Android platform tools and libraries, Android Studio latest stable release for integrated development environment and Gradle (version 8.5) to build automation, dependency management, and optimized Android builds. It is available on the Android and iOS platforms via the Google Play Store and Apple App Store. The software architecture follows a modular, service-oriented design, with clearly defined functional components for user authentication, content management, notification delivery, analytics tracking, and data synchronization. All intervention content will be finalized and frozen prior to participant enrollment. No modifications, additions, or removals of educational content will be made during the 12-month trial period.

#### App Version Control

A single, version-controlled release of *Pehli Ghiza* will be used for all intervention participants to ensure consistency of exposure. Any updates during the trial, if required, will be limited strictly to technical maintenance (eg, bug fixes or compatibility updates) that does not alter content, messaging, or functionality. All such changes will be documented with version numbers, timestamps, and change logs. This approach is adopted to minimize the risk of confounding due to midtrial intervention modifications.

#### Intervention Access Control

Although *Pehli Ghiza* is publicly available, access will be restricted through study-specific onboarding, registration, and backend activation, provided only to participants randomized to the intervention arm to mitigate contamination. The control group will not be able to access the app, as participants will not be provided with login credentials. Further, recruitment and follow-up procedures for both arms will be conducted separately to minimize cross-arm information exchange. App usage logs will be monitored to detect unauthorized access, and any crossover will be documented and addressed through intention-to-treat and sensitivity analyses.

### Control

#### Standard-of-Care F2F Counseling

**Figure 4.**
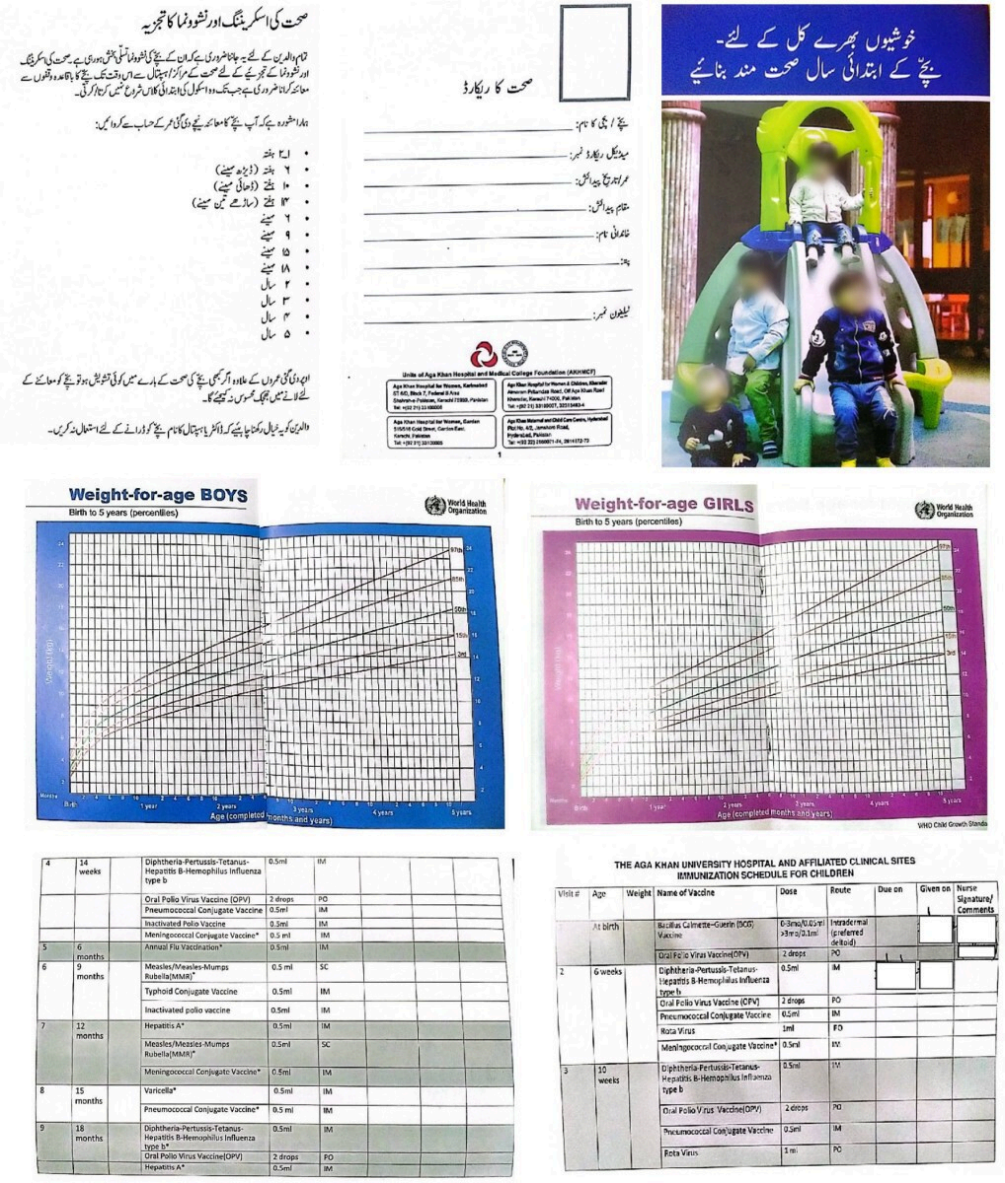
Standard of care is a patient information booklet that provides general information about child development, nutrition, growth, and vaccination record.

Participants in both the control and intervention arms will receive F2F counseling by the RA at each follow-up visit as part of routine standard-of-care. To ensure consistency across study arms and follow-up visits, the RA will provide counseling using the AKUH patient education booklet (Figure 4) as a standardized counseling script. The booklet includes information on maternal nutrition, breastfeeding, IYCFPs, child health, growth, and immunization. Each counseling session will be approximately 20 minutes in duration and will not include additional reinforcement beyond standard service delivery.

#### Counseling Standardization and Fidelity Monitoring

The RA will undergo standardized training sessions for adherence to uniform counseling procedures and scripts. This will minimize variation in counseling content, delivery, and interaction across participants. To further ensure fidelity, periodic supervisory reviews and checklist-based monitoring of counseling sessions will be conducted throughout the study.

### Outcome Measures

#### Outcome Assessment and Schedule

The outcome indicators are derived from WHO IYCF indicators [49,50]. Outcome data will be collected using structured interviewer-administered questionnaires and anthropometric assessments at predefined follow-up visits. The schedule of outcome measurements is presented in Table 2.

**Table 2.** Schedule of enrollment, interventions, and assessments of participants as per SPIRIT (Standard Protocol Items: Recommendations for Interventional Trials).

Study steps	Baseline	Postallocation						
		Birth	6 wk	10 wk	14 wk	6 mo	9 mo	12 mo
Enrollment and allocation								
Eligibility screen	✓^a^							
Informed consent	✓							
Randomization	✓							
Allocated intervention								
mHealth^b^ coaching	mHealth coaching will occur continuously across the study period with the SoC^c^, not just at specific time points							
Face-to-face counseling	✓	✓	✓	✓	✓	✓	✓	✓
Participants characteristics								
Maternal	✓							
Infant		✓						
IYCF^d^ knowledge, attitude, and breastfeeding self-efficacy								
Baseline (at enrollment)	✓							
Midline (14th wk)					✓			
Endline (12th mo)								✓
Study outcome								
Early initiation of breastfeeding		✓						
Exclusive breastfeeding^e^			✓	✓	✓	✓^e^		
Continuation of breastfeeding at 1 y			✓	✓	✓	✓	✓	✓
Introduction of age-appropriate complementary feeding^e^						✓^e^	✓	
Minimum dietary diversity						✓	✓	✓
Minimum meal frequency						✓	✓	✓
Minimum acceptable diet						✓	✓	✓
Anthropometry		✓	✓	✓	✓	✓	✓	✓
Acute respiratory infection			✓	✓	✓	✓	✓	✓
Acute diarrhea			✓	✓	✓	✓	✓	✓
Visit to emergency room			✓	✓	✓	✓	✓	✓
Hospitalization			✓	✓	✓	✓	✓	✓
mHealth usability								
Assessment								✓

a ✓ indicates step conducted or outcome measured at the specific time point.

b mHealth: mobile health.

c SoC: standard of care.

d IYCF: infant and young child feeding.

e The 6-month assessments of exclusive breastfeeding and age-appropriate complementary feeding will serve as the primary outcome.

#### Primary Outcomes

The primary end points of the study are (1) EBF at 6 months of infant age and (2) introduction of AACF [49,50] at 6 months of infant age.

In addition, EBF will also be assessed at the 6th, 10th, and 14th weeks, while CF practices will also be assessed at 9 months of infant age. The repeated assessments at additional time points will be used for longitudinal analyses to evaluate trends and sustainability of feeding practices over time. Operational definitions of the primary outcome measures are provided in Table 3.

**Table 3.** Operational definitions of primary and secondary outcome measures.

Outcome	Operational definitions
Primary outcomes [49,50]	
Exclusive breastfeeding	Proportion of infants who are fed exclusively with breast milk up to 6 months of age
Introduction of age-appropriate complementary feeding	Proportion of infants who are introduced to solid, semisolid, or soft foods at 6 mos of age
Secondary outcomes	
Infant and young child feeding practices [49,50]	
Early initiation of breastfeeding	Proportion of children put to the breast within 1 hour of birth
Minimum dietary diversity	Proportion of children 6‐12 months of age who receive food from 4 or more food groups
Minimum meal frequency	Average number of daily meals for children 6‐12 months of age, who receive solid, semisolid, or soft foods
Minimum acceptable diet	Composite variable based on meal frequency and dietary diversity
Mean duration of breastfeeding	Average duration an infant is breastfeeding for 12 months
Continued breastfeeding at 1 y	Proportion of children 12 months of age who are still on breast milk
Child health	
ARI^a^	Short, rapid breathing that is chest-related and/or difficult breathing that is chest-related. Percentage of children up to 1 year of age with symptoms of ARI at any time in the last 2 weeks preceding the follow-up
Acute diarrhea	Passage of 3 or more loose or liquid stools per day (or more frequent passage than is normal for the individual). Percentage of children up to 1 year of age with symptoms of diarrhea at any time in the last 2 weeks preceding follow-up
Visit to the emergency room	Infant brought to the emergency room for management of any acute illness in past 2 weeks.
Hospitalization	Infant admitted to the hospital for diagnosis or management of any acute illness in past 2 weeks.
Stunting (height-for-age) [51]	Children whose height-for-age *z* score is below −2 SD from the median of the reference population used in the WHO^b^ Child Growth Standards [41]
Wasting (weight-for-height) [51]	Children whose weight-for-height *z* score is below −2 SD from the median of the reference population used in the WHO Child Growth Standards [41]
Overweight (weight-for-height) [51]	Children whose weight-for-height *z* score is more than 2 SD above the median of the reference population used in the WHO Child Growth Standards [41]
Underweight (weight-for-age) [51]	Children whose weight-for-age *z* score is below (−2 SD) from the median of the reference population used in the WHO Child Growth Standards
Breastfeeding self-efficacy [52]	A standard 14-item tool adapted and translated into Urdu language to determine self-efficacy in breastfeeding, rated on a 5-point Likert scale
mHealth^c^ usability [53]	A questionnaire to assess experience of mHealth application based on 6 domains: design, interface, content, coaching, perception, and personal benefit, rated on a 5-point Likert scale

a ARI: acute respiratory infection.

b WHO: World Health Organization.

c mHealth: mobile health.

#### Secondary Outcomes

IYCFP-related secondary outcomes include EIBF, continued breastfeeding at 1 year, mean duration of breastfeeding, MDD, MMF, and MAD [49,50]. Child health–related secondary outcomes include acute respiratory infection, acute diarrhea, visits to the emergency room, hospitalization, stunting (height-for-age) [51], wasting (weight-for-height) [51], overweight (weight-for-height) [51], underweight (weight-for-age) [51], breastfeeding self-efficacy [52], and mHealth usability [53]. Operational definitions of the secondary outcome measures are provided in Table 3.

### Follow-Up

Outcome assessments are aligned with the national Expanded Programme on Immunization schedule to maximize participant adherence. These will be collected at birth; at the 6th, 10th, and 14th weeks; and at the 6th, 9th, and 12th months of infant age by the RA. At each follow-up visit, participants in both study arms will receive F2F counseling. Follow-up windows of ±2 weeks will be permitted to accommodate participant availability and logistical constraints. Phone call reminders will be given prior to their appointments, with the option to reschedule, where needed. When in-person visits would not be feasible, counseling and outcome data will be collected through telephone follow-up or during subsequent health facility visits to minimize loss to follow-up.

### Retention Strategy

The RA will remain engaged with all study participants fortnightly to maintain contact, confirm continued participation, and address any study-related or technical concerns. Participants in the intervention arm will be inquired regarding application functionality and, if necessary, provided assistance with troubleshooting or reinstallation of the app to ensure uninterrupted access. For participants in the control arm, the RA will maintain equivalent contact to minimize differential attention across study arms. Follow-up windows will remain flexible to accommodate participant availability, and alternative contact modalities will be used where needed. Attrition will be continuously monitored, and reasons for withdrawal will be documented. If attrition exceeds anticipated levels, this will be addressed analytically through intention-to-treat analyses, supported by sensitivity analyses using appropriate methods for missing data.

### Data Collection

Demographic information, including maternal age, education, employment status, maternity leave entitlements, income, ethnicity, area of residence, and obstetric history, will be collected electronically at the time of enrollment through a pretested structured questionnaire in Urdu. These factors will be examined in the analysis as potential confounders to assess their influence on IYCFP adherence and intervention outcomes. Study outcomes and anthropometric measurements will be recorded at each follow-up visit by the RA using standardized structured questionnaires. Participant knowledge, attitude, and breastfeeding self-efficacy will be collected at recruitment, the 14th week, and 12 months of infant age. The study schedule is reported according to the SPIRIT (Standard Protocol Items: Recommendations for Interventional Trials) 2013 statement.

### Data Management Plan

All study data will be collected electronically with predefined range and logic checks within the database to detect out-of-range values, inconsistencies, and missing data at the point of entry and during routine data cleaning. Version control procedures will be applied to all study datasets. Any modifications will be documented with version numbers, timestamps, and descriptions of changes. An audit trail will be maintained to automatically record all data creation, modification, and access events, including user identity and the date of change.

### Data Monitoring

All study data will be monitored in real time through a centralized digital dashboard. The Research Scholar will do day-to-day data monitoring, ensure protocol adherence at the study sites, and track participant adherence and withdrawals. The Principal Investigator will conduct fortnightly supervisory meetings, review study progress, and arrange periodic audits to ensure data quality and protocol compliance. RAs will receive refresher training periodically to address any identified quality issues.

### Adverse Events Monitoring, Reporting, and Management

Although the intervention is behavioral and noninvasive, anticipated harms include digital fatigue, psychological stress related to health messaging, technical issues such as app malfunction, and potential concerns related to data privacy. All potential adverse events will be actively monitored throughout the study period. Participants will be encouraged to report any discomfort, distress, or technical difficulties to the RA. All reported adverse events will be documented, reviewed, and categorized by type and severity. Serious or unexpected adverse events will be referred for consultation. Appropriate technical or supportive actions will be taken as needed, including temporary suspension of app notifications or technical troubleshooting.

### Data Security and Confidentiality

Participant data will be stored in a centralized, encrypted database hosted on secure servers, with role-based access controls to ensure that only authorized study personnel (the Principal Investigator and Research Scholar) can access identifiable information. Data transmission between the mobile app, backend servers, and web portal is protected using industry-standard encryption protocols (HTTPS). Personally identifiable information is stored separately from usage and engagement data to enhance privacy protection. The platform complies with established data protection and confidentiality principles, including secure authentication, password protection, and audit logging.

### Study Reporting Guidelines

This RCT protocol is reported according to the SPIRIT guidelines.

### Statistical Analysis

#### Data Analysis Plan

Baseline characteristics will be summarized by randomized group using frequencies and percentages for categorical variables (eg, education, socioeconomic status, area of residence, and employment status) and mean (SD) or median (IQR) for continuous variables (eg, maternal age and parity), as appropriate. Normality of continuous variables will be assessed using the Shapiro-Wilk test. Baseline comparability will be evaluated using chi-square or Fisher exact tests for categorical variables and independent-samples *t* tests or Mann-Whitney *U* tests for continuous variables.

All primary and secondary analyses will follow the intention-to-treat principle, including all participants as randomized. Primary outcomes (EBF and AACF at 6 mo of infant age) are binary and will be analyzed using regression models appropriate for dichotomous outcomes. The results will be expressed as adjusted risk ratios or odds ratios with 95% CIs. Treatment effects will be reported with corresponding 95% CIs and 2-sided *P* values. Repeated measures of EBF (at the 6th, 10th, and 14th weeks) and CF practices will be analyzed using mixed-effects regression models with random intercepts to account for within-participant correlation and fixed effects for treatment group, time, and group-by-time interaction.

Secondary outcomes that are binary, including EIBF, continued breastfeeding at 1 year, MDD, MMF, and MAD, will be analyzed using similar logistic regression approaches. Continuous secondary outcomes, including mean duration of breastfeeding and child anthropometric *z* scores (height-for-age, weight-for-height, and weight-for-age), will be analyzed using linear regression or linear mixed-effects models, with results reported as mean differences and 95% CIs.

Breastfeeding self-efficacy and mHealth usability will be measured using validated multi-item Likert-scale instruments. Composite scale scores will be generated by summing or averaging item responses and analyzed as continuous outcomes using linear or mixed-effects linear regression models, as appropriate. Distributional assumptions will be assessed, and robust standard errors or nonparametric methods will be applied in sensitivity analyses if substantial departures from normality are observed.

The extent and patterns of missing data will be examined. If more than 10% of outcome data are missing, multiple imputation using chained equations will be performed under a missing-at-random assumption. Imputed results will be compared with complete-case analyses in sensitivity analyses. Prespecified subgroup analyses will assess effect modification by maternal education, employment status, and parity through inclusion of interaction terms. All analyses will be conducted using Stata software (StataCorp), with statistical significance set at .05.

#### Patient and Public Involvement Statement

Patients and the public are not involved in the design, recruitment, or implementation of this study. Study participants will be informed about the results through awareness seminars conducted at the study site.

### Ethical Considerations

The study approval has been granted by the institutional Ethical Review Committee (Ref 2022-3424-20757, received on February 6, 2025) and the National Bioethics Committee for Research (Ref 4‐87/NBCR-1114/23/204, received on August 15, 2025). In the event of any protocol deviations or amendments, the Ethical Review Committee will be notified and approval will be sought. Written informed consent will be obtained from all eligible participants prior to enrollment by the RA at recruitment. Participants will be provided with detailed information regarding the study objectives, procedures, potential risks and benefits, data usage, and confidentiality protections. They will also be given adequate opportunity to discuss the study, ask questions, and clarify any concerns before providing consent. Participants will be informed that participation is voluntary and that they may withdraw at any time without any effect on their routine care. Consent will be documented electronically and via signed consent forms before installation of the mHealth app.

## Results

The study has been approved by the Ethical Review Committee of the Aga Khan University and the National Bioethics Committee. Participant recruitment started on February 2, 2026. As of May 14, 2026, we have recruited 230 participants. Follow-ups and outcome assessments are expected to be completed by June 2027, and analysis is expected to be completed by September 2027. We expect the results to be published by December 2027.

## Discussion

### Expected Contributions and Future Implications

This study is expected to provide evidence regarding the effectiveness of a culturally tailored mHealth intervention in improving EBF and AACF during the first year of infant age, while repeated longitudinal assessments may provide insights into the sustainability of feeding behaviors over time. Previous mHealth interventions conducted in Nigeria, Nepal, Indonesia, and Pakistan have primarily demonstrated improvements in maternal IYCF knowledge, breastfeeding awareness, and user satisfaction [23-27]; however, evidence regarding sustained behavioral outcomes remains inconsistent [28-30]. Systematic reviews from LMICs further highlight substantial heterogeneity and limited longitudinal evidence regarding sustained IYCF outcomes [31-34]. This study addresses these gaps by evaluating a theory-informed, culturally tailored mHealth intervention through an RCT for sustained feeding practices during the infant’s first year of life in Pakistan, where rigorous evidence on digital nutrition interventions remains limited. If found effective, the intervention may offer a scalable and potentially cost-effective strategy for strengthening maternal and child nutrition services in Pakistan and similar LMIC settings. Study findings will be disseminated through peer-reviewed publications, scientific conferences, and engagement with health care providers, policymakers, and public health organizations to support evidence-informed digital health programming and nutrition policy development.

### Limitation

We acknowledge limitations in our study. First, participation is limited to literate women who own smartphones, which may restrict generalizability. However, this criterion is necessary to ensure valid exposure to the digital intervention and to evaluate its feasibility under real-world conditions. Second, despite restricted onboarding and backend access controls, some degree of information sharing between intervention and control participants may occur in community settings. Such contamination will be monitored through usage analytics and addressed analytically using intention-to-treat and sensitivity analyses. Third, behavioral outcomes may be subject to recall or social desirability bias. To mitigate this, standardized questionnaires, repeated assessments, and objective app usage metrics will be used where feasible. Fourth, sustained engagement with the app over the 1-year intervention period may decline due to digital fatigue or competing demands. Automated reminders and regular RA contact are incorporated to support adherence and maintain engagement. Fifth, blinding of participants and research staff is not feasible due to the nature of the intervention, which may introduce observer or reporting bias. However, this risk will be minimized through standardized outcome assessment procedures, structured questionnaires, RA training, and blinded data analysis using coded group allocation. Finally, the study assesses intervention effectiveness over a 1-year period and does not evaluate long-term sustainability or scalability. Findings will inform future studies designed to assess broader implementation and longer-term impact.

### Strengths

This study’s strengths include its rigorous 2-arm parallel-group randomized controlled design, the first randomized trial in Pakistan, to evaluate the efficacy of an mHealth app targeting EBF and age-appropriate IYCFPs, thus addressing a critical national evidence gap. The intervention is theory-driven, culturally tailored, and aligned with WHO and UNICEF guidance, enhancing relevance and acceptability. Longitudinal follow-up from late pregnancy through the infant’s first year allows assessment of both early initiation and sustained feeding behaviors. Objective monitoring of intervention exposure through backend analytics strengthens internal validity beyond self-reported measures, while provision of standard-of-care F2F counseling in both arms enables estimation of the incremental benefit of the mHealth intervention. Finally, the use of widely available smartphone technology enhances scalability and policy relevance within Pakistan’s expanding digital health ecosystem.

## Supplementary material

10.2196/93181 Checklist 1 SPIRIT checklist.

## Abbreviations

AACF: age-appropriate complementary food
AKUH: Aga Khan University Hospital
CF: complementary feeding
CONSORT: Consolidated Standards of Reporting Trials
EBF: exclusive breastfeeding
EIBF: early initiation of breastfeeding
F2F: face-to-face
IYCF: infant and young child feeding
IYCFP: infant and young child feeding practice
LMICs: low- and middle-income countries
MAD: minimum acceptable diet
MDD: minimum dietary diversity
mHealth: mobile health
MMF: minimum meal frequency
RA: research assistant
RCT: randomized controlled trial
SDK: software development kit
SPIRIT: Standard Protocol Items: Recommendations for Interventional Trials
WHO: World Health Organization
